# The Cellular Impact of the ZIKA Virus on Male Reproductive Tract Immunology and Physiology

**DOI:** 10.3390/cells9041006

**Published:** 2020-04-18

**Authors:** Raquel das Neves Almeida, Heloisa Antoniella Braz-de-Melo, Igor de Oliveira Santos, Rafael Corrêa, Gary P. Kobinger, Kelly Grace Magalhaes

**Affiliations:** 1 Laboratory of Immunology and Inflammation, Department of Cell Biology, University of Brasilia, 70910–900 Brasilia, Distrito Federal, Brazil; raquel.unb.bio@gmail.com (R.d.N.A.); heloisa.antoniella@gmail.com (H.A.B.-d.-M.); igorosantosbio@gmail.com (I.d.O.S.); rafaelcorrea31@gmail.com (R.C.); 2 Département de Microbiologie-Infectiologie et d’Immunologie, Université Laval, Quebec City, QC G1V 4G2, Canada; Gary.Kobinger@crchudequebec.ulaval.ca; 3 Centre de Recherche en Infectiologie du CHU de Québec-Université Laval, Quebec City, QC G1V 4G2, Canada

**Keywords:** Sertoli cells, Leydig cells, ZIKA virus, arboviruses, infertility

## Abstract

Zika virus (ZIKV) has been reported by several groups as an important virus causing pathological damage in the male reproductive tract. ZIKV can infect and persist in testicular somatic and germ cells, as well as spermatozoa, leading to cell death and testicular atrophy. ZIKV has also been detected in semen samples from ZIKV-infected patients. This has huge implications for human reproduction. Global scientific efforts are being applied to understand the mechanisms related to arboviruses persistency, pathogenesis, and host cellular response to suggest a potential target to develop robust antiviral therapeutics and vaccines. Here, we discuss the cellular modulation of the immunologic and physiologic properties of the male reproductive tract environment caused by arboviruses infection, focusing on ZIKV. We also present an overview of the current vaccine effects and therapeutic targets against ZIKV infection that may impact the testis and male fertility.

## 1. Introduction

The testis is a reproductive gland that is part of the internal structures of the male reproductive tract (MRT) and is involved in spermatogenesis and steroidogenesis. Each testis is composed of a tangle of tubes, the seminiferous ducts. These ducts are formed by Sertoli cells (SCs) and the germinal epithelium, which is responsible for ensuring protection and nutrition to accurate spermatogenesis. Leydig cells (LCs) are found in the testis interstitium, adjacent to the seminiferous tubules. LCs promote steroidogenesis through the secretion of male sex hormones, especially testosterone, responsible for the development of male genital organs and secondary sexual characters [1,2].

The testis is considered an immune-privileged organ [3]. This is essential to ensure the immunogenic germ cell protection against immune system activation during spermatogenesis. This is mainly provided by the combination of a local immunosuppressive environment and systemic immune tolerance [4,5,6]. It has long been assumed that the blood–testis barrier (BTB) constitutes the main mechanism of the immune-privileged status of the testis [7]. In addition to BTB and anatomical impairment of external cells’ and molecules’ entrance to testis, SCs also provide anti-inflammatory mediator secretion aiming to maintain the tolerogenic microenvironment [8]. However, many local immune modulators, including macrophages, dendritic cells (DCs), natural killer cells (NKs), mast cells and T-lymphocytes, contribute to the intercommunication among testicular components [9,10,11,12].

The testis is commonly exposed to pathogens derived from blood, trauma, or through the genitourinary tract. To protect itself against all these pathogens, the testis also needs the ability to overpower immune privilege. This is achieved by inducing local innate immune responses [3]. Even counting this frontline protection, some pathogens have an immune scape mechanism that leads to infection and persistence in the MRT. Reproductive tract infections (RTI) can be caused by bacterial, parasitic, and viral pathogens [13]. RTI promoted by viral infections are notorious, as shown by the World Health Organization (WHO) in 2006, which estimated that 500 million people live with genital herpes, 300 million women have human papillomavirus (HPV), and approximately 240 million people suffer from chronic hepatitis B [14]. In 2016, the WHO also estimated that over 17 million people are living with HIV on antiretroviral therapy. However, the number of HIV-positive cases is increasing worldwide [15].

Some diseases can persist a long time in human semen. Ebola [16], Zika virus (ZIKV) [17], HIV [18], and 27 other types of viruses that contaminate humans have been found in semen and testis for differing periods [19]. Despite the knowledge that various types of viruses can be found in semen, their sexual transmission capacity is still poorly understood. Some of these are not considered sexually transmitted diseases because this route is not the main form of contagion. However, ZIKV has already been confirmed by the WHO to have sexual transmission (World Health Organization, 2016) and considered to be the first arbovirus reported to be associated with sexual transmission [20,21]. Due to this fact, attention is being turned to the possibility that other arboviruses may be present in the MRT. Compared to ZIKV, the literature regarding this effect is scarce, and the available data suggests that arbovirus sexual transmission is a relevant point of concern. The presence of ZIKV in the male genital tract and its ability of sexual transmission leads to unanswered questions such as (1) has the ZIKV a tropism for any specific cell in the male reproductive system?, (2) what features may favor the ZIKV persistence in testicles when compared to other arboviruses?, (3) can the spermatozoa harbor the virus?, (4) how long does the virus remain viable in the male genital tract?, (5) how can the prolonged presence of ZIKV in the male genital tract cause infertility?, (6) is this ZIKV-induced testicular damage reversible? Based on these questions, it is clear the importance of continuing to investigate the role of ZIKV in the male reproductive system. In addition, a vaccine against ZIKV may be the best way to protect the population from infection, and control the disease and its consequences. The vaccination should protect against future and possible damage to the male genital tract, avoiding fertility-related problems. Therefore, in this review, we will address recent findings of ZIKV infections in the MRT, focusing on cellular mechanisms, immune and physiological responses, and the ability to other arboviruses to remain in the testicle.

## 2. Male Reproductive Tract (MRT) and Cellular Composition of Testis

**Figure 1:**
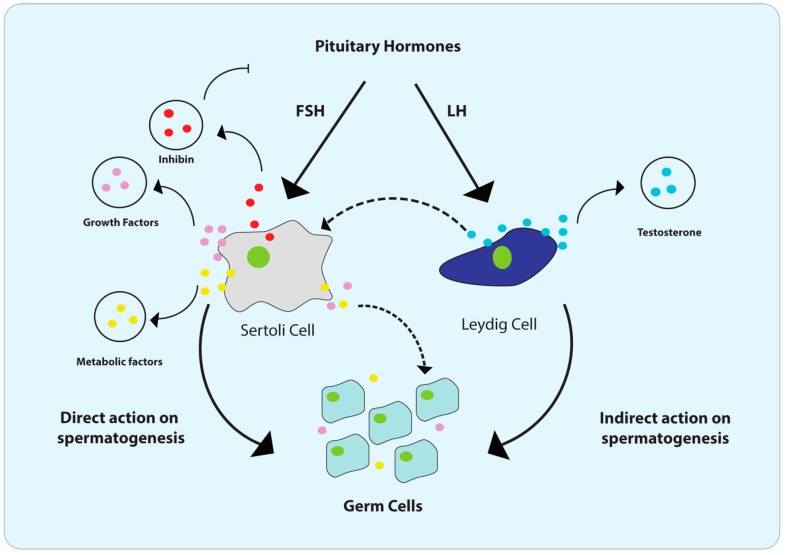
Cellular crosstalk during normal spermatogenesis. Pituitary hormones follicle-stimulating hormone (FSH) and Luteinizing Hormone (LH) have an important role in spermatogenesis. FSH leads to Sertoli cell proliferation stimulating the release of inhibin. LH triggers the production of the testosterone by Leydig cells, which can stimulate the release of metabolic and growth factors by Sertoli cells and indirectly trigger spermatogenesis in germ cells. Metabolic factors, such as lactate and growth factors, can directly drive the spermatogenesis in germ cells. Oppositely, inhibin produced by Sertoli cells can inhibit FSH release by pituitary gland acting as a negative feedback regulation.

The MRT is composed of sexual organs that play a major role in the male germ cells (or sperm) production. It has mainly consisted of a pair of testicles that are specialized for androgen hormones and gamete production, an intromittent organ that is responsible for depositing sperm on the female reproductive tract and finally a couple of sexual accessories ducts and glands vital for sperm maturation, nutrition, and storage [22,23]. The different cell types that compound these tissues of MRT maintain crosstalk that allows the production of viable sperm in the testis (Figure 1). Once the homeostasis of the system is broken, this process is impaired and the fertility capacity is altered [24].

The testis is composed of interstitial LCs located between blood vessels and the seminiferous tubules, where sperm is produced [25]. LCs secrete androgens that participate in conjunction with pituitary hormones (gonadotropin) in germ cell development [26]. On the other hand, seminiferous tubules include the germ cells, which give rise to spermatozoa through a series of differentiation steps and the somatic SCs [23]. Somatic SCs are essential not just for testes formation but are one of the major conductors of gametogenesis [27]. The immunological infiltrate in the interstitial compartment of the normal testis, especially resident macrophages, is also important to directly influence testicular microenvironment [28].

The seminiferous tubules present an anatomical barrier that impairs the blood-derived factor input to the testicular microenvironment without any regulation [29]. BTB is the main factor responsible for regulating the paracellular transit of molecules. The BTB is the result of tightly cellular junctions of adjacent SCs in addition to epithelial and myeloid cell interaction [27]. The presence of this barrier creates separated compartments and protects against immunological infiltrate that could lead to testicular inflammation [30]. The unbalanced inflammatory response can disrupt BTB integrity, causing non-specific entry of harmful molecules that impair sperm cell maturation. Nevertheless, cytokine release is a regulatory factor during spermatogenesis in controlled levels [31]. It is important to emphasize that the transit of immune cells is not fully blocked once leukocytes have been reported in normal testicular surroundings, especially close to spermatozoa. Macrophages are the most abundant immunological cells that reside in seminiferous tubules environment and present an important role of immune-surveillance of the germ cell development process.

In the testis, macrophage characterization demonstrated novel functions associated with germ cell development, androgen hormone production, and maintenance of a homeostatic microenvironment [28]. Studies have shown that there are two distinct macrophages populations in testicular surroundings: the CD163^−^ newly arrived macrophages and CD163^+^ resident testicular macrophages. The CD163^+^ macrophages are polarized to the type 2 macrophage (M2) profile that constantly secretes anti-inflammatory molecules, such as interleukin-10, in the seminiferous tubules acting as a protective component against sperm cell damage [32]. On the other hand, newly arrived CD163^−^ macrophages are related to the inflammation maintained in the seminiferous tubules. These cells secrete higher levels of pro-inflammatory cytokines, such as interleukin-1β and tumoral necrosis factor-α, and present a higher expression of nitric oxide synthase (iNOS), demonstrating a pro-inflammatory profile, a key characteristic of type 1 macrophage (M1) [32]. The communication of these cells with LCs, SCs, and germ cells seems to be important in the development process that leads to sperm production. Macrophages are being called as true sentinels of testis function [28]. In addition, Matusali and colleagues have found evidence of ZIKV infection of the testicular CD163^+^ resident macrophages [33]. ZIKV-induced cell death of CD163^+^ resident macrophages could also contribute to the inflammation in testis.

In addition to macrophages, other immunological cells are found in testicular surroundings. DC are antigen-presenting cells found in testicular interstitial spaces and represent a minor population of leukocytes in the testis. DCs induce activation and differentiation of lymphocytes in response to allo-antigens and minimize autoimmune response by tolerating T-cells to auto-antigens under physiological conditions [12]. Other immune cells, including NKs, T-cells, and CD4^+^CD25^+^ regulatory T-cells (Tregs), are also found [10]. Besides, mast cells are present in a great number regarding immune cell populations in the testis during puberty [34]. However, the functions of these cells in the maintenance of testicular immune-privileged sites remain unclear [35].

The process of male mature gamete production is called spermatogenesis and consists of the intense proliferation and subsequent differentiation of spermatogonial stem cells to spermatozoa [25]. The crosstalk between constituent cells of the testis is essential in this process [36,37], once the energy source of gametes during differentiation depends on the lactate that is provided by SC. On the other hand, glucose capitation depends on androgen hormone signalization provided by LC, as well as pituitary hormones, insulin sensibility, and paracrine communication [36,37]. This is one of the central reasons that explains why altering testicular cell metabolism impairs the production of viable sperm [38]. Important factors as epigenetics (including miRNA regulatory activity), growth factors, and cytokine release also influence the process in the quantity and quality of the sperm [39,40].

Spermatogenesis starts in puberty, long after the perinatal self-tolerance process. For this reason, sperm cells contain a new repertory of proteins that present a great potential of activating an immune response, leading to autoimmunity [3]. Studies have shown that activation of T-lymphocytes and the production of specific antibodies against sperm cells are related to the infertility process. It was also reported that the production of intense pro-inflammatory cytokines is related to loss of BTB integrity and loss of viable sperm, leading to infertility [30,41]. Avoiding this massive activation, the testis presents a unique tolerogenic microenvironment, making the organ immune-privileged, and protecting mature gametes against the immune-cell-induced death and inflammation.

The immune-privileged microenvironment is essential for the viability of sperm cells and maintenance of testis function while, at the same time, serving as a site for the persistence of infections due to the tolerogenic surrounding. Microorganisms coming from blood or urogenital infections enter into a testicular environment and disrupt tissue homeostasis, leading to activation of local immune system [3]. This process triggers testicular inflammation and may alter tissue metabolism, signalization, cellular function, and leading to impaired spermatogenesis and spermiogenesis [42,43]. Many pathogens have been shown to cause male infertility by many mechanisms, induced inflammation being the key for most of them.

## 3. Flavivirus and ZIKV Features

The *Flavivirus* genus is composed by viruses of small single-stranded RNA. The flaviviruses can cause mild symptoms, such as fever, pain, and cutaneous rash but also covers severe disturbances, such as encephalitis, neurological complications, and hemorrhagic fever [44]. Flaviviruses are arthropod-borne pathogens typically transmitted by mosquitoes or tick vectors and are related to significant mortality and morbidity worldwide [45]. Members with clinical relevance of this genus include Dengue virus (DENV), Yellow Fever virus (YFV), Japanese Encephalitis virus (JEV), West Nile virus (WNV) and ZIKV. The geographic distribution of flaviviruses and the diversity of arthropod vectors make them of great interest for epidemiological surveillance. Moreover, the easy entry and adaptation of these viruses in new environments make this genus relevant to extensive research and experimental studies [44].

**Figure 2:**
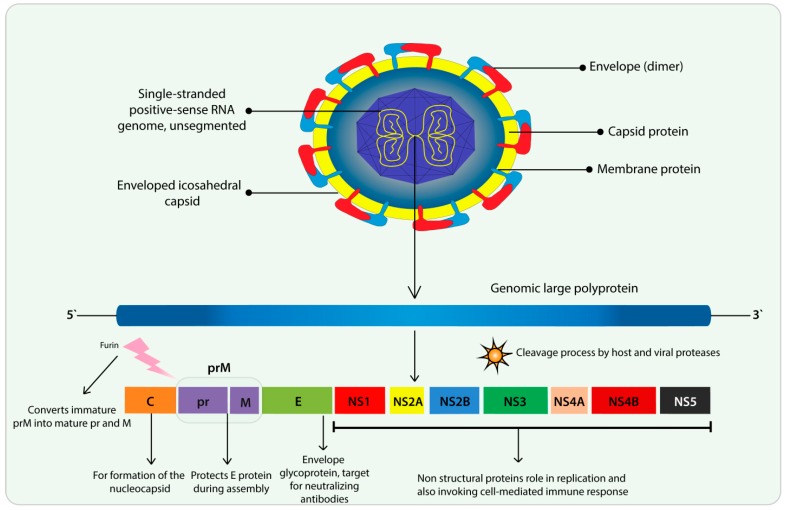
Zika virus (ZIKV) structure and features. ZIKV is an enveloped positive-sense single-stranded RNA virus composed by envelope, capsid, membrane protein, and single-stranded positive-sense RNA. The lower part represents the polyprotein which is cleaved by viral and cellular proteases four structural proteins: capsid (C), envelope (E), precursor membrane (prM), and membrane (M) and seven non-structural proteins (NS1, NS2A, NS2B, NS3, NS4A, NS4B, and NS5). During infection, the ZIKV E proteins bind to host cell receptors and the viral particle is endocytosed. The E proteins enable the fusion of the virus with the endosomal membrane, leading the release of the genomic RNA into the host cell cytoplasm. The translation of the RNA genome occurs in the endoplasmic reticulum. The RNA is translated as a single polypeptide chain encompassing all the viral proteins: C-prM-E-NS1-NS2A-NS2B-NS3-NS4A-NS4B-NS5.

ZIKV is a vector-borne flavivirus belonging to the *Flaviviridae* family, with two main lineages: the African and the Asian lineage [46]. It is an enveloped virus measuring about 50 nm in diameter with a non-segmented, positive single-stranded ribonucleic acid (RNA) genome (Figure 2). The genome is made up around of 11 kb with a single open reading frame that codes structural proteins: Capsid (C), Envelope (E), precursor membrane (prM); and non-structural proteins (NS1, NS2A, NS2B, NS3, NS4A, NS4B, and NS5) [47] (Figure 2).

The first ZIKV isolate was identified in primates in 1947 in Uganda Protectorate in a program for surveillance of yellow fever in primates [48]. The first human infection was reported in 1954 in Nigeria; for decades, ZIKV cases were restricted to Africa and Asia [49]. Since 1954, several outbreaks with increasing number cases have been reported worldwide [50,51]. The last outbreak was documented in 2015 in America, which was the largest epidemic ever described of ZIKV affecting more than 20 countries [52,53]. In 2016, WHO considered ZIKV a public health emergency of international concern [20].

ZIKV has different pathways of transmission. The ZIKV transmission in humans was firstly reported through bites of infected *Aedes aegypti* or *Aedes albopictus* mosquito [54]. However, the virus was identified and isolated from seventeen different *Aedes mosquitos* species, *Culex quinquefasciatus*, *Culex perfuscus*, *Mansonia uniformis*, *Anopheles coustani,* and *Anopheles gambiae* mosquitoes [55,56,57,58,59]. Another important fact about ZIKV transmission became apparent during the 2015 outbreak, when several cases of ZIKV vertical transmission were identified from an infected mother through the placenta to the fetus and sexual transmission (male-to-female; female-to-male; male-to-male) [60]. This novel mode of ZIKV transmission in humans had never been reported before in flavivirus infection [60,61,62]. ZIKV was the first arbovirus detected in human semen [63]. While needing more consistent evidence about the ZIKV transmission, these findings suggest the complexity of ZIKV dynamics transmission [64,65].

## 4. ZIKV on Male Reproductive Tract

**Figure 3:**
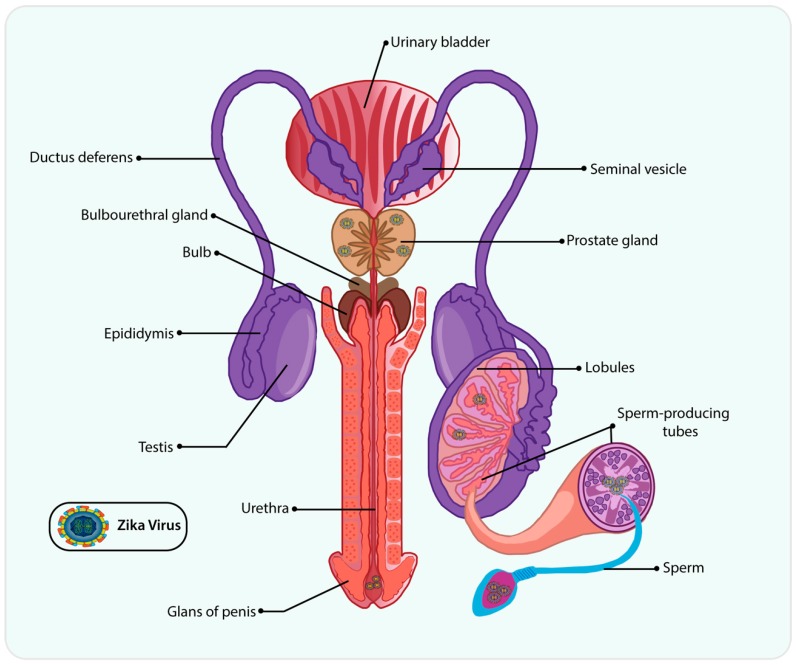
ZIKV reservoir in the male reproductive tract. ZIKV has been found in several portions of the male reproductive tract, including the prostate gland, testicle, epididymis, and seminiferous tubules. ZIKV-infected men have presented prostatitis, hematospermia, and microhematospermia. ZIKV RNA has been detected in semen from ZIKV-infected men and sexual transmission is an important route of contagious ZIKV. Some testicular cells are susceptible to ZIKV infection, such as spermatogonia, primary spermatocytes, Sertoli cells, and spermatozoa. Moreover, ZIKV can infect and replicate in mature sperm, leading to male infertility.

The male reproductive system includes the penis, scrotum, testicles, epididymis, vas deferens, prostate and seminal vesicles (Figure 3). Recent studies have demonstrated the presence of ZIKV RNA in semen, as well as in male and female reproductive tracts, indicating the occurrence of the sexual transmission [66]. The first sexual transmission became evident in 2011, and many cases have supported the idea of one potential transmission pathway [62]. Moreover, ZIKV could be detected in semen six months after infection in negative ZIKV serum from a patient [67]. Similarly, ZIKV RNA was detected in semen in symptomatic and asymptomatic-infected patients [68,69,70]. A case report showed ZIKV RNA presence in total semen and also in the sperm fraction used in assisted reproductive technology up to 112 days after infection [71]. Taken together, all these data indicate that infected men can be a potential reservoir for sexual transmission, even a long time after the infection [72].

In a mouse model, ZIKV sexual transmission was recently characterized, showing that epididymal epithelial cells and leukocytes should be the main source of ZIKV RNA shedding [73]. ZIKV can persist and replicate in MRT [74]. In cases of ZIKV infection, is it known that SCs can support a high level of ZIKV replication [75,76]. In the early stages of infection, ZIKV suppresses cell growth, cell proliferation, and dysregulation of germ cell–SC junction signaling [77]. ZIKV downregulated the secretion of inhibin B, a hormone mostly produced by SCs [78]. Strange and colleagues demonstrated a unique cross-talk between ZIKV infection and SC immune response, which in the course of infection, the viral persistence was associated with activation of canonical pro-inflammatory pathways. That includes the upregulation of genes of the human leukocyte antigen (HLA) class I, pro-inflammatory genes such as interleukin-23 subunit alpha (IL23A) and lymphotoxin beta (LTB), NF-kappa-B-epsilon (NFKBIE), IL6, STAT1, STAT2, and IFN [77].

The IFN response is a strong key in the innate immune response against virus dissemination in testicles. Two animal models of *Mus musculus* species, susceptible to ZIKV infection, are important for understanding the pathogenesis of this virus. These models are A129 and AG129 mice, both immunocompromised mice. A129 mice do not have the receptor for interferon type I (IFN α/β). AG129 mice do not have the receptor for interferon type I and II (IFN α/β/γ) [79]. IFNAR^−/−^ mice are one of the best mice models for ZIKV susceptibility studies [80]. Siemann and colleagues have shown that in the first hours of infection, ZIKV does not induce IFN-α in SC, but it presents a modest induction after 48 and 72 h of infection. However, high levels of pro-inflammatory cytokines such as interleukin-1α (IL-1α), IL-1β, IL-6, IL-8, and TNF-α were found in the supernatant of infected SC, and in the chemokines such as RANTES (CCL5), fractalkine (CX3CL1), and IP-10 (CXCL10). These levels increased significantly 72 h after infection. Although SCs generate a strong immune response against ZIKV, the virus can persist in the male reproductive tract for a long time [81].

The TAM receptor, AXL, promotes the ZIKV entrance in SCs and contributes negatively to the antiviral states of SCs [82]. SCs are one type of cell that expresses high levels of TAM receptors, TGF-β expression, and activin-A to maintain the immune regulation in the seminiferous tubules. SCs play an important role in testicular physiology, creating a BTB and contributing to the nourishment of the spermatozoa. This cellular physiology and ZIKV modulation can develop an important factor that may lead to the establishment of viruses in this organ. Other cell types in the testicle can support the ZIKV infection, such as testicular fibroblast, germ cells, and spermatocyte [43,83].

LCs and testicular macrophages are part of the first line of defense in the seminiferous tubules [84]. LCs are not highly susceptible to ZIKV infection in mice models, but more studies in humans are necessary. However, LCs are the main source of testosterone in testis, and during ZIKV infection, the levels of testosterone are significantly modulated [78]. Testicular macrophages are infected by ZIKV [33], and the infection promotes an increase of mRNA transcript levels of the IFN-α and IFIT1 genes, inducing the secretion of pro-inflammatory cytokine TNF-α, IL-1α, and IL-8 and chemokines, such as GRO, IP-10, and monocyte chemoattractant protein 1 (MCP-1). These inflammatory mediators are correlated with the possibility that ZIKV infection can compromise SC barrier integrity [81]. ZIKV does not modulate the expression of tight junction proteins (TJPs). The virus can cross BTB efficiently and persist in abluminal side seminiferous tubules by the induction of adhesion molecules expression such as VCAM-1, which facilitates the adhesion of immune cells, compromising BTB permeability [81].

In spermatogonia, the infection can promote cell death, leading to the destruction of seminiferous tubules and triggering male infertility by damaging the male reproductive system [75]. Low sperm counts are observed in patients infected with ZIKV [69,85]. Several studies have shown the effect of the ZIKV infection promoting genital damage, modulation of testicular immunity leading to orchitic and viral replication, promoting a long infection establishment. ZIKV does not affect only the testes. In mice and monkey models, ZIKV infection causes acute and chronic prostatitis [86]. Male rats infected with the Mexican ZIKV strain presented a significant decrease in testicle size compared to uninfected rats. Testicle atrophy may have occurred due to decreased testosterone levels in cells infected with this virus [87].

Several studies have shown alterations in mature sperm infected by ZIKV [85,88]. Such findings may also be an additional indication that ZIKV reduces male fertility. Furthermore, it is important to evaluate sperm banks regarding the presence of ZIKV-infection in donors due to the implications for assisted reproduction.

**Figure 4:**
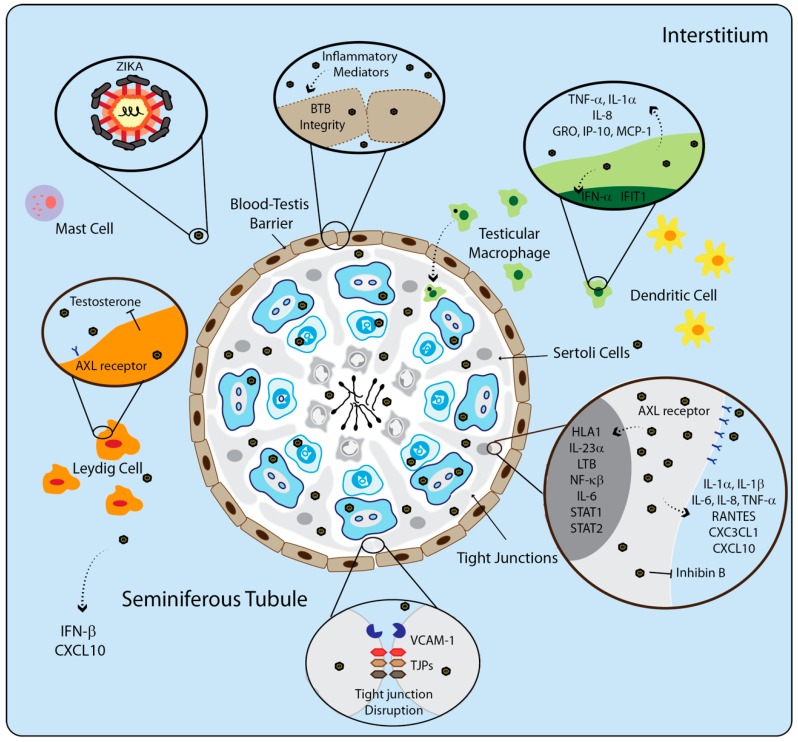
Testicular cells infection by ZIKV. ZIKV infection can cause serious physiological, immunological, and endocrine damage in the testes, impairing spermatogenesis. ZIKV can infect several cells in the male reproductive tract. Leydig cells are less susceptible to the infection when compared to other cells in the male reproductive tract. Testosterone, the main hormone produced by Leydig cells, is modulated by ZIKV, impairing the endocrinological function. Testicular macrophage is infected by ZIKV, triggering upregulation of IFN-α, IFIT1, TNF-α, IL-1a and IL-8, GRO, IP-10, and MCP-1. Inside the seminiferous tubule, Sertoli cells have high expression levels of AXL receptors, which is used by ZIKV to invade cells. Sertoli cells support high levels of ZIKV replication, and the infection promotes the upregulation of genes related to antigen presentation (HLA-1), proinflammatory cytokines (lymphotoxin-beta LTB, IL-6, IL-23a) and transcription factor related to inflammation (NF-kb, STAT1, and STAT2). The release of proinflammatory cytokines such as IL-1α, IL-1β, IL-6, IL-8, TNF-α, and chemokines such as RANTES, CXC3CL1, and CXCL10 in SCs is also promoted by the infection. These molecules can promote the chemoattraction of more immunological cells and lead to an inflammatory profile, impairing efficient spermatogenesis. Inhibin-B, produced predominantly by SCs, can control follicle-stimulating hormone (FSH) secretion and is downregulated by ZIKV infection. ZIKV increases the expression of VCAM-1 in SCs which can facilitate the immune cells adhesion. Inside the seminiferous tubules, ZIKV can infect spermatogonia, primary spermatocytes, and mature spermatozoa.

Therefore, ZIKV is capable of entering the testicular microenvironment, disrupting cellular metabolism, altering testicular physiology, and activating an intense immune response, which can result in severe testicular damage and infertility. A better understanding of how ZIKV affects the regulation of cell survival pathways and the testicle physiology can help evaluate pathogenesis and may be used for vaccine studies to identify intervention strategies (Figure 4).

## 5. The Immune System of Testis during Viral Infection

MRT requires a homeostatic microenvironment for viable germ cell production and nutrition. The crosstalk between SCs and LCs is fundamental to spermatozoa development [89,90].

In the testicular surroundings, an important immunological component maintains a proper environment for spermatogenesis, turning the testis into an immune-privileged organ [91]. Once MRT homeostasis is broken, spermatogenesis key steps are impaired and inflammation can be trigged. Many pathogens have shown to infect and persist in the MRT [3,26,84]. Testicular abnormalities, infertility, or sexual transmission are some of the major consequences of pathogen persistence in the MRT. Considering the important findings regarding ZIKV RNA detection in the semen, the scientific community has turned their attention to the possibility that other flaviviruses promote similar effects [92]. Once their detection becomes proven, the possibility of sexual transmission or impaired spermatogenesis is another important factor to be explored. Preliminary studies about this have provided us with information on a possible threat derived from different flaviviruses in the MRT. Nevertheless, this question is far from clear and molecular mechanisms still under investigation.

Some studies have been reported flavivirus infection in the MRT [19]. The viral load could be found for some of them, and the presence of leukocytes in the semen suggests an inflammatory process caused by the infection. Salam and colleagues found viruses from several families in the semen, including *Adenoviridae*, *Filoviridae*, *Flaviviridae*, *Herpesviridae*, and *Retroviridae* [19].

DENV is a considerably more common flavivirus than ZIKV, and the knowledge about DENV effects in the testis is scarce. The first case report linking DENV infection to MRT modulation was published in 2011 [93]. In this report, scrotal and penile edema was a rare complication associated with DENV infection. However, the mechanism by which this edema was formed was not evaluated; neither could DENV be detected in penile fluids. Currently, there is no data reporting if testicular abnormalities could be trigged by DENV-associated inflammation in MRT. In 2018, two controversial publications raised questions about the possible impact of DENV in the MRT. The first one demonstrated that DENV RNA was not detected in the semen of five confirmed patients during the acute infection [94]. The second one is a case report released a few days later, demonstrating that DENV was detected in the semen of an infected man 37 days after the related symptoms. The report demonstrated DENV RNA in the cellular fraction, suggesting the possibility of sexual transmission [95]. New evidence of DENV sexual transmission was published in 2019, where a case report from Spain detected the viral RNA in the semen of two men who were partners [96]. Only one of the men had contact with a DENV endemic area and his partner presented the symptoms a few days after the first one. This is the first evidence of DENV sexual transmission. Nevertheless, clinical trials aiming to concisely respond to this question are underway and may be published soon (Clinical Trial Identifier: NCT03612609).

In 2018, a case report was published regarding YFV RNA detection in the semen and urine of a Brazilian man in the convalescent phase of the disease [97]. The integrity and infectivity of the viral particles were accessed and confirmed in the report. This strongly suggests that this virus can be sexually transmitted once it is capable of maintaining infective parameters, although no data are available confirming the capability of YFV persistence and impact in the MRT, or sexual transmission associated with the infection.

Zheng and colleagues showed that the JEV infection induces inflammation of pig testicles by activating RIG-I/NF-kB pathway signaling [98]. This also leads to orchitis, which is a type of chronic inflammation in the testes caused by viral or bacterial infections, associated with pain, swelling, along with blood and swelling in prostate ejaculate [81,99]. Testes infection with JEV showed a differential production of pro-inflammatory cytokines, such as IL-1β, IL-6, IL-8, chemokine RANTES, and TNF-α, as well as an increased presence of NS5 (non-structural protein of the virus), RIG-I, TLR3 and -7 [98]. Smith and colleagues showed that a 43-year-old patient presented signs of encephalitis and orchitis caused by WNV [100]. In this report, histological sections showed lymphocyte, SCs, and interstitial multinucleated cells infiltrate, as well as marked thickening of the basement tubular membranes and absence of spermatogenesis, an indication of atrophy. Numerous foci of dense chronic interstitial inflammatory infiltrate and necrotic cell death was observed in the seminiferous tubules [100].

DENV, YFV, and JEV are classified both as arbovirus and flavivirus and present major clinical relevance within these groups. Nevertheless, another important virus that compounds arbovirus group but is a member of a distinct family, presents important findings regarding MRT infection. For this reason, an analysis of the available data for this arbovirus is relevant and will also be explored in this section.

The Chikungunya virus (CHIKV) is a small, enveloped, single-stranded positive-sense RNA virus that belongs to *Alphavirus* genus and *Togaviridae* family. Chikungunya is a vector-borne disease, also transmitted by the bites of mosquitoes from the *Aedes* genus, mainly *Ae. aegypti* and *Ae. albopictus*, causing arthritis or arthralgia, which is accompanied by fever and rash [101]. CHIKV RNA has been detected in semen and urine, as reported in a case published in 2016. This study showed a patient presenting CHIKV and DENV (type 3) dual infection, in which only CHIKV was detected [102] in both the acute and convalescence phases of the disease, within 30 days after symptoms. Thereby, it is important to emphasize that CHIKV presents tropism and cytotoxic effects on monocyte-derived macrophages [103], which can be later recruited to testicular microenvironment [32]. In this context, macrophages are being identified as a possible source of CHIKV RNA in the testis, acting as a testicular trojan horse. However, more studies are necessary to verify this hypothesis [102].

Numerous questions related to viruses infection in MRT remain to be answered. The long-term effects of persistent infection for several flaviviruses in male reproductive function, as well as production and fertility of spermatozoa need to be investigated. Importantly, in the case of ZIKV, cryptorchidism, hypospadias and micropenis have been reported in newborn infants of infected mothers [104], although its prevalence is unknown. An effect of arboviruses infection in male fertility will only be fully understood in long-term epidemiological studies and suitable animal model experiment design.

## 6. ZIKV Vaccines and Treatment to Improve the Host Response in the MRT

Sexual transmission of ZIKV and the viral persistence in the MRT are the strongest challenges for outbreaks control, vaccines, and antiviral drug development [105,106,107]. The impact of ZIKV infection in the population leads to a significant global efforts to develop vaccines. Spectacular progress has been made in ZIKV vaccine development, and several strategies have been proposed to increase vaccine protection in immune-privileged organs [105,108,109,110,111,112].

Antibody usage has shown a promising strategy to protect ZIKV in the testicle. Some subclasses of immunoglobulin (IgG) can cross the BTB [113]. The administration of human antibodies to DENV E-dimer epitope (EDE1-B10) 3 days after infection was able to reduce the viral load in testis, reducing the inflammation and preserving sperm count. The protection is not effective for the long duration [114]. Further studies in this area have explored the pathogenesis pathways and the host cellular response, suggesting potential targets to develop vaccines, including DNA-based vaccines. DNA-based vaccines, and live attenuated ZIKV have shown testicular protection against infection, avoiding atrophy, damage, and male infertility [74,115].

The combined strategies of DNA-based vaccines and live attenuated ZIKV vaccines demonstrated efficacy when used in a single-dose in A129 mice. This vaccination promotes the complete prevention of testicle infection, injury, and oligospermia [116]. Another live-attenuated ZIKV vaccine, which presents one deletion in the 3′ untranslated region of the ZIKV genome (ZIKV-3′UTR-LAV), presented protection after a single vaccination in mice and non-human primates. This protection was evaluated for preventing mother-child vertical transmission and the prevention of testicle damages [117].

DNA-based vaccination of recombinant chimpanzee adenovirus type 7 (AdC7) expressing ZIKV M/E glycoproteins presents high efficacy in a single vaccination. AdC7-M/E induced a potent neutralizing antibody in immunocompetent and immunodeficient mice and full protection against ZIKV-induced testicular damage [118]. Another DNA-based vaccine, encoding ZIKV pre-membrane and envelope (prME) in pVAX vector, protected mice completely against ZIKV, promoting protection in testes and sperm and decreasing viral persistence in MRT [115]. Moreover, this vaccine was also effective in reversing mouse infertility [119].

A few drugs against ZIKV have also been proposed and may have an impact on testicles [106,120,121]. Recently, Z2 an amphipathic peptide derived from the stem region of ZIKV envelope protein was reported to inhibit vertical ZIKV transmission in a mouse model and reduce viral load in the testicle and epididymis. This was also reported to reduce pathological damage while improving sperm quality [122]. Simanjuntak and colleagues demonstrated that ZIKV-infected testicles presented progressive damage with a significant oxidative microenvironment, with high levels of reactive oxygen species, nitric oxide, glutathione peroxidase 4 and pro-inflammatory cytokines as IL-1β, IL-6, and G-CSF. They proposed the use of the antioxidant ebselen (EBS) to prevent the sexual transmission of the virus and to improve host testicular immune response [123].

## 7. Conclusions

ZIKV can infect and persist in testicular somatic and germ cells, as well as, spermatozoa, leading to cell death and testicular atrophy. ZIKV has also been detected in semen samples from ZIKV-infected patients. This has huge implications for human reproduction. DNA-based vaccination and/or live attenuated ZIKV vaccines showed high efficacy against MRT damage induced by ZIKV and are a very prominent therapeutic tool to prevent male infertility caused by ZIKV.

It is important to note that, often, no evident testicular inflammatory response is usually observed against ZIKV infection in testes, with normal testicular morphology and hormone production remaining unaffected after ZIKV infection. This indicates that ZIKV can remain quiescent in the testes, acting as a trojan horse, and maintaining asymptomatic ZIKV sexual transmission. The better understanding of the mechanisms that mediate the cellular impact of the ZIKV on MRT, regulating testicular immune and physiological responses, is the key factor to the correct design of efficient anti-ZIKV therapeutic strategies to prevent male infertility caused by ZIKV.
